# Hantaviruses in the Americas and Their Role as Emerging Pathogens

**DOI:** 10.3390/v2122559

**Published:** 2010-11-25

**Authors:** Brian Hjelle, Fernando Torres-Pérez

**Affiliations:** 1 Center for Infectious Diseases and Immunity, Department of Pathology, University of New Mexico Health Sciences Center, MSC08 4640, Albuquerque, New Mexico 87131, USA; E-Mail: ftorresp@gmail.com; 2 Biology Department, University of New Mexico, MSC03 2020, Albuquerque, New Mexico 87131, USA

**Keywords:** hantavirus, hantavirus cardiopulmonary syndrome, emergence, pathology, epidemiology

## Abstract

The continued emergence and re-emergence of pathogens represent an ongoing, sometimes major, threat to populations. Hantaviruses (family Bunyaviridae) and their associated human diseases were considered to be confined to Eurasia, but the occurrence of an outbreak in 1993–94 in the southwestern United States led to a great increase in their study among virologists worldwide. Well over 40 hantaviral genotypes have been described, the large majority since 1993, and nearly half of them pathogenic for humans. Hantaviruses cause persistent infections in their reservoir hosts, and in the Americas, human disease is manifest as a cardiopulmonary compromise, hantavirus cardiopulmonary syndrome (HCPS), with case-fatality ratios, for the most common viral serotypes, between 30% and 40%. Habitat disturbance and larger-scale ecological disturbances, perhaps including climate change, are among the factors that may have increased the human caseload of HCPS between 1993 and the present. We consider here the features that influence the structure of host population dynamics that may lead to viral outbreaks, as well as the macromolecular determinants of hantaviruses that have been regarded as having potential contribution to pathogenicity.

## 1. Introduction

Emerging pathogens cause new or previously unrecognized diseases, and among them, emerging zoonotic diseases are a major concern among scientists studying infectious diseases at different spatial and temporal scales [1,2]. Changes in biotic and abiotic conditions may alter population disease dynamics and lead to the emergence of zoonotic infections [3–6]. During the last decades, several outbreaks of emerging and re-emerging viral pathogens have occurred, affecting both purely-local and worldwide/pandemic involvement of human populations. Among the conspicuous examples are influenza A, Ebola virus, hepatitis C virus, severe adult respiratory distress (SARS), coronavirus, and human immunodeficiency virus, which challenge prevention and control measures of public health systems [7]. In the Americas, the recent outbreak of pandemic influenza A subtype H1N1 became a major target for control due to its rapid spread, and uncertainties in virulence and transmissibility, yet vaccine availability was limited when significant activity occurred in advance of the traditional influenza season [8]. However, in the last century outbreaks of several viral-related diseases have emerged or re-emerged involving arenaviruses and dengue viruses, and more recently, hantaviruses, and the expansion of the geographic range of West Nile virus. Among zoonotic diseases, small mammals are hosts of several pathogenic RNA viruses, especially Arenaviridae and Bunyaviridae: *Hantavirus* [9–11].

Hantavirus infections became a concern in the Americas after the description of an outbreak of acute respiratory distress occurred in the Four Corners area in 1993 [12]. The newly recognized disease, hantavirus cardiopulmonary syndrome, HCPS (or hantavirus pulmonary syndrome), was linked to infection by the newly-discovered Sin Nombre virus (SNV), and the rodent *Peromyscus maniculatus* (deer mouse) was identified as the reservoir [13]. However, hantavirus infections have a much longer history. A review of ancient Chinese writings, dating back to approximately 960 AD, revealed descriptions closely resembling hemorrhagic fever with renal syndrome (HFRS), the syndrome caused by Old World hantaviruses [14]. During the twentieth century, cases of acute febrile disease with renal compromise were described from several Eurasian countries and Japan, often in association with military engagements [15]. HFRS as a distinct syndrome, however, was first brought to the attention of western medicine in association with an outbreak that occurred among United Nations troops during the Korean conflict between 1951 and 1954, where more than 3,200 soldiers were afflicted [16]. It took more than two decades until the etiologic agent, Hantaan virus (HTNV), was isolated from the striped field mouse *Apodemus agrarius,* detected in part by the binding of antibodies from patient serum samples to the lung tissues of healthy, wild-caught field mice [17,18]. The virus was later found to represent the type species of a new genus *Hantavirus* of the family Bunyaviridae, although it was later apparent that the first hantavirus to be isolated was the shrew-borne Thottapalayam virus [19]. The categorization of hantaviruses as belonging to the family Bunyaviridae is due in part to the consistent presence of three RNA genomes that are circularized *in vivo* as a result of the presence of terminal complementary nucleotides that help fold the genome into a “hairpin” morphology, first described for the Uukuniemi phlebovirus [19,20]. Table 1 is a list of the predominant, serologically distinct pathogenic hantaviruses. Many other named genotypes are described, but such other pathogenic forms are generally closely related to Andes or, in some cases, *Sin Nombre virus*.

**Figure 1:**
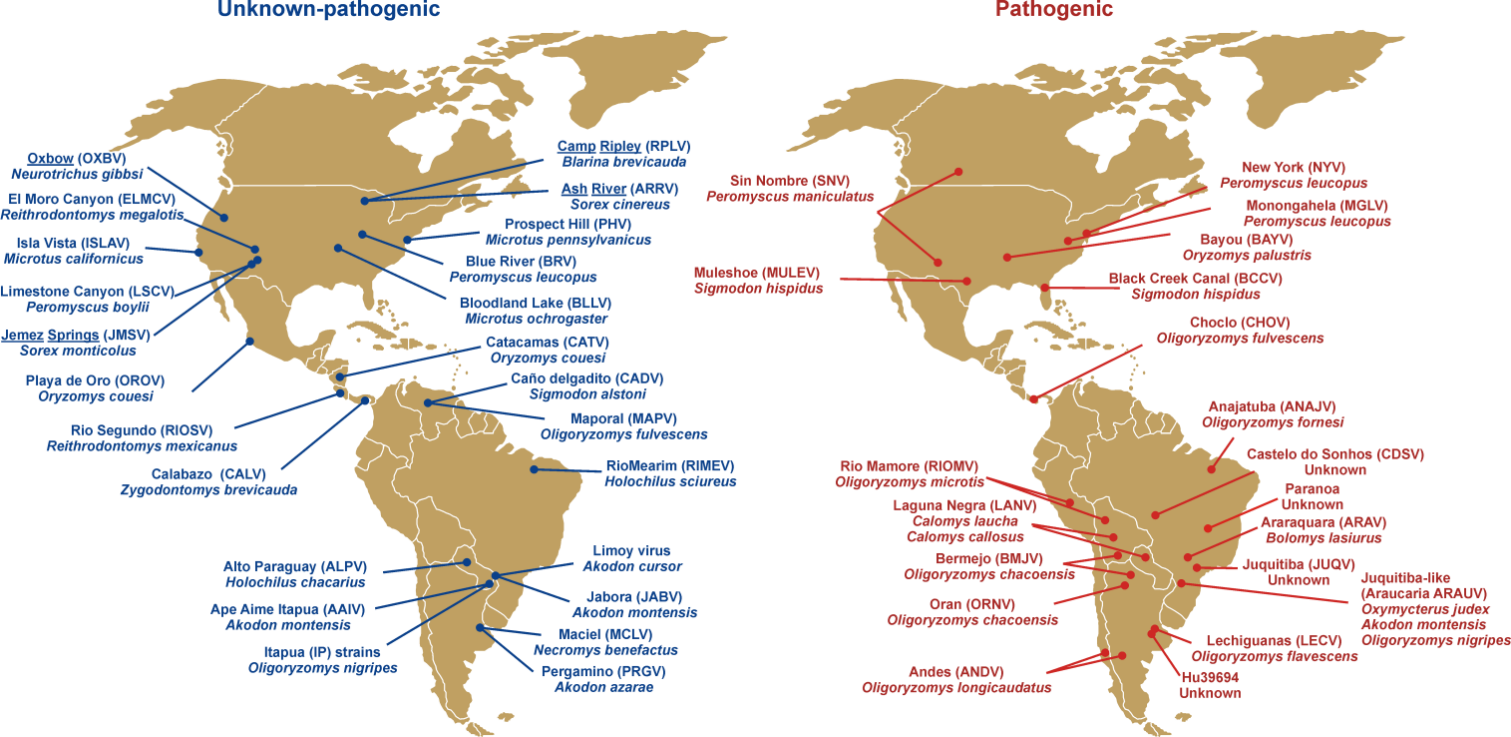
Map of hantavirus genotypes in the Americas reported to be non-pathogenic or of unknown pathogenicity (blue) and pathogenic (red) for humans. Hantaviruses reported in Soricomorpha (shrews and moles) are underlined.

Hantavirus discovery continues at a rapid pace. After the identification of SNV in 1993, approximately 43 genotypes have been reported in the Americas alone (Figure 1), with 20 of those associated with clinical cases of HCPS. Most hantaviruses worldwide have been identified in murid or cricetid rodents of the subfamilies Murinae, Arvicolinae, Neotominae, and Sigmodontinae as their main reservoir, but the discovery of hantaviruses in insectivores (Soricidae, Talpidae) on at least three continents [21,22] shows that the genetic diversity of hantaviruses is far higher than expected.

## 2. Molecular Features and Cell Interactions

Member viruses of the family Bunyaviridae, including those of the genus *Hantavirus* are spherical, enveloped viruses of 100–200 nm diameter with a tripartite helical nucleocapsid. The hantavirus genome is composed of three negative sense single stranded RNA segments: (i) The large (L) segment of approximately 6,500 nucleotides (nt) encodes a RNA-dependent RNA polymerase of ∼2,160 amino acids (aa); (ii) The middle (M) segment is 3,700-nt long, and encodes the envelope glycoprotein precursor (GPC), which is processed into two transmembrane glycoproteins, Gn (652 aa, formerly “G1”) and Gc (488 aa, previously “G2”) through a cotranslational proteolytic mechanism in the endoplasmic reticulum [23–26]; (iii) The small (S) segment length varies from ∼1,700 to 2,100 nt, and encodes an RNA-binding nucleocapsid (N) protein that ranges between 428 and 436-aa for hantaviruses.

### 2.1. Envelope Glycoproteins

During virus maturation, the precursor form GPC is processed using a membrane-bound protease into Gn and Gc, a cleavage that occurs, and appears to be signaled, after the conserved peptide signal WAASA at the C-terminal of Gn [24]. Although the two proteins can be expressed independently through transfection, they can be retained in the wrong cellular compartment (ER or aggresome); they thus must be co-expressed to allow them stability so that the two can be assembled correctly in the Golgi [25,27–29].

A number of activities and properties have been identified for the hantavirus envelope glycoproteins, including some features that are suspected to be involved in the pathogenicity of the disease-causing serotypes, a possibility that has engendered experimental attention. The glycoproteins are the known or presumed ligands for at least two distinct cellular receptors, the β3 integrin chain and decay accelerating factor, or DAF [30,31]; with gC1qR/p32 also identified as another potential entry receptor [32]. Comparisons with the tick-borne encephalitis virus E protein, led Tischler *et al.* to consider the Gc glycoprotein as a potential class II fusion protein, perhaps imparting fusion activity to the virion, and this hypothesis has gained support in other studies [33,34].

Additional activities have been identified with, or claimed to be related to, Gn. For many of these studies, an underlying premise has held that there are differences between the glycoproteins of “pathogenic” hantaviruses relative to viruses in the genus that are dubbed to be “non-pathogenic”. While it is true that it has not yet been possible to link Prospect Hill virus (PHV) to human disease, the absence of evidence for its pathogenicity should perhaps not be equated with the evidence of its absence. One might only consider that the level of disease (e.g., lethargy, fever, proteinuria, and azotemia) associated with infection of nonhuman primates by PHV is not significantly different from that recorded for nonhuman primate models using the known-pathogen Puumala virus (PUUV) [35,36]. For the purpose of this discussion we will presume that apathogenic hantaviruses are indeed apathogenic.

While some studies have suggested that Gn glycoproteins are directed more rapidly into the ubiquitin-proteosome pathway than are apathogenic forms, others have interpreted differences in the handling of Gn glycoproteins across hantavirus species by the ubiquitin-proteosomal system as independent of pathogenicity [37–39]. Some investigators have directed their efforts toward identifying a differential capacity, either kinetic or in absolute magnitude, in the ability of pathogenic and apathogenic hantaviruses to elicit an interferon response in cells. One premise that emerges is that apathogenic forms would tend to induce an earlier innate response that would render it more likely that the virus would be quickly cleared or rendered less competent in its replication so as to blunt any pathological response in the host [40–42]. The anti-hantavirus innate response can in some cases be attributed to viral interaction as a ligand of TLR-3, but not in others, and in endothelial cells, it appears not to require more than the viral particle itself, even when introduced in replication-incompetent form [43,44]. Proteins and mRNAs prominently induced by hantaviruses include MxA and IFIT-1 (ISG-56) and others including some with known or suspected anti-viral activity. Those hantaviruses, often highly pathogenic strains, that fail to induce a potent antiviral response, are suspected or presumed to have a (more) potent interferon-pathway antagonism mechanism relative to other viruses, a mechanism that acts positively to prevent an effective innate response from forming, at least early in infection [42,45]. Yet some instances are reported wherein highly pathogenic hantaviruses, such as SNV, are also able to induce expression of interferon-stimulated gene mRNAs, even very early in infection, with ISG proteins, as expected, taking longer to appear in the cell [44]. Anti-interferon activities have also been attributed to the NSs protein that may be elaborated in cells infected by serotypes that encode this protein [46]. Other investigators have examined the activities of hantavirus glycoproteins and other proteins that might themselves directly affect some aspects of the pathogenic progression associated with hantavirus infection of humans, such as vascular permeability changes. While early attempts to directly cause increases in permeability of endothelial monolayers with viral particles or viral infection were largely disappointing, hantaviruses have been identified as adversely affecting endothelial migration over substrata and in potentiating VEG-F-induced endothelial permeability [47,48].

### 2.2. The Hantavirus Nucleocapsid Protein

The shorter (≈50-kD) nucleocapsid or N protein is a structural component of the viral nucleocapsid, along with the genomic viral RNA segments. As an RNA-binding protein that engages the hairpin termini of the genomic segments with high affinity [49,50], it limits the access of the RNA to host nucleases and helps to render viral replication a closed process within the cytoplasm. It also acts as a peripheral membrane protein, as does the L protein [51], an activity that could play a role in its presumed, but not yet demonstrated function as matrix [52]. Until recently, it had not been appreciated that N has a wide variety of other activities, some of which can be linked, not only to fundamental requirements of replication, but also to the interference with an array of the intracellular processes of the normal cell. Thus, an interaction between the amino terminus of the hantavirus N protein and the cellular protein Daxx has been proposed, with the suggestion of potential pro-apoptotic consequences [51]. N is also reported to interact with actin microfilaments, and the SUMO-1 protein [53,54]. Using reporter-gene based assays, Connie Schmaljohn and her colleagues have reported that Hantaan virus’ nucleocapsid protein has an inhibitory role in inflammatory responses mediated by NF kappa B (NF-κB). The effects on NF-κB expression appeared to be confined to prevention of its nuclear translocation after its attempted activation with lipopolysaccharide, LPS [55]. In the cytoplasm of infected cells, N protein can be found in cellular P bodies where it sequesters and protects 5′ caps. It may locate the caps through its interaction with DCP1, a key constituent of P bodies. During hantavirus infection, the viral RNAs become concentrated in P bodies, through their interaction with N and DCP1. The N protein demonstrates preferential protection of mRNAs engineered to prematurely terminate their encoded protein in comparison to native mRNAs [56].

N protein has been increasingly linked to viral replication and translation, sometimes in previously unanticipated ways. It is among a growing family of diverse viral proteins that can serve as a nonspecific “RNA chaperone”, an activity that should facilitate the L polymerase’s access to vRNA for transcription and replication, in that it can transiently dissociate misfolded RNA structures [57]. Some of N protein’s effects on translation might not immediately be recognized to be adaptive in nature. It can replace the entire EIF4F translational initiation complex, simultaneously presenting the ribosome with a replacement for the cap-binding activity of eIF 4E, binding to the 43S pre-initiation complex as does eIF 4G, while replacing the helicase activity of eIF 4A, which is presumed to be needed to dissociate higher-order RNA structure [56,58]. These three factors normally work together to achieve translational initiation. In P bodies, N protein’s ability to bind at high affinity to capped native cellular oligoribonucleotides, along with its activity in protecting capped RNAs from degradation likely facilitates the access of capped oligonucleotides for use in transcriptional initiation by L polymerase (“cap snatching”).

Trafficking of N for viral assembly: Classically, N protein in infected cells appears to be clustered or particulate in nature, with a heavy concentration at a single perinuclear location, widely considered to be the Golgi [27]. The N proteins of hantaviruses are found in association with particulate fractions, and confocal microscopy and biochemical-inhibitor studies have shown that N tracks along microtubules but not with actin filaments [52]. The ultimate destination for N, for its assembly into viral particles is the Golgi, and it traffics there via the endoplasmic reticulum-Golgi intermediate complex (ERGIC), also known as vesicular-tubular cluster [52]. A dominant negative inhibitor, dynamitin, associated with dynein-mediated transport, reduced N’s accumulation in the Golgi. Later studies suggested that the specific dependence on microtubular transport is specific to Old World hantaviruses such as HTNV, but that the New World hantavirus ANDV is instead associated with actin filaments [59]. However, recent data indicates that microtubular transport is indeed utilized for the New World hantavirus SNV [60].

## 3. Pathology and Diagnosis

Hantavirus diseases of man have long been suspected of having an immunopathogenic basis in part because of their relatively long incubation period of 2–3 weeks and the observed temporal association between immunologic derangements and the first appearance of signs and symptoms of hantavirus illness. HFRS and HCPS share many clinical features, leading many investigators to consider them to be, in essence, different manifestations of a similar pathogenic process, differing mainly in the primary target organs of disease expression (Table 2). The pathogenesis of hantavirus infections is the topic of a continuously-updated review in the series UpToDate [61].

By the time symptoms appear in HCPS, both strong antiviral responses, and, for the more virulent viral genotypes, viral RNA can be detected in blood plasma or nucleated blood cells respectively [63,64]. At least three studies have correlated plasma viral RNA with disease severity for HCPS and HFRS, suggesting that the replication of the virus plays an ongoing and real-time role in viral pathogenesis [65–67]. Several hallmark pathologic changes have been identified that occur in both HFRS and HCPS. A critical feature of both is a transient (∼ 1–5 days) capillary leak involving the kidney and retroperitoneal space in HFRS and the lungs in HCPS. The resulting leakage is exudative in character, with chemical composition high in protein and resembling plasma.

### 3.1. Pathology

The continued experience indicating the strong tissue tropism for endothelial cells, specifically, is among the several factors that make β3 integrin an especially attractive candidate as an important *in vivo* receptor for hantaviruses. It is likely that hantaviruses arrive at their target tissues through uptake by regional lymph nodes, perhaps with or within an escorting lung histiocyte. The virus seeds local endothelium, where the first few infected cells give rise, ultimately, to a primary viremia, a process that appears to take a long time for hantavirus infections [62,63].

By the time that secondary viremia emerges, the agents of the more severe forms of HFRS and HCPS have begun to achieve sufficient mass as to induce, through PAMP-PRR interactions and other means, the expression of proinflammatory cytokines [64]. For HCPS, that expression favors the pulmonary bed and lymphoid organs, yet, for unknown reasons, spares the retroperitoneum and, in general, the kidney. In HFRS the situation is reversed, and yet it is often not appreciated that the expected preferential tissue tropism of HFRS-associated viruses and their HCPS-associated counterparts for the renal and pulmonary beds, respectively, is not as one would predict through the manifestations of the two diseases.

Local elaboration of inflammatory and chemotactic mediators is considered to be a requirement for the development of systemic disease symptoms, with those abnormalities sometimes culminating in shock and death. Yet it is not hypoxemia, due to the prominent pulmonary edema, that leads to death in most fatal cases of HCPS, but rather intoxication of the heart by as-yet-undefined mediators that leads to the low cardiac output state and the associated shock syndrome [64,65]. It is tempting to speculate that mediators produced in the lung in connection with the inflammatory infiltrate can percolate through the coronary circulation with minimal dilution in HCPS, a disadvantageous consequence of the close anatomic juxtaposition of the two organs. Thus, at least three classes of potential mechanisms, some overlapping and all certainly nonexclusive of the others, could be presumed to underlie the pathogenesis of HCPS. These include:

(1) Innate immune mechanisms. The nature of interactions between hantavirus pathogen-associated molecular patterns (PAMP) with the pattern recognition receptors (PRR) of susceptible endothelial cells are beginning to be clarified. The prototypical HTNV appears to be recognized by TLR-3 [43]. Such an infection has consequences such as increased expression of HLA-DR in dendritic cells [66] and differentiation of monocytes toward dendritic cells [67].

(2) Direct viral effects. The observed correlation between viral load and disease severity leaves the possibility open that hantavirus particles or RNA can themselves have toxic effects on cells or on signaling. Some investigators have favored direct viral toxicity, acting through the inhibition of endothelial cell barrier function, as an explanation for much of the capillary leak, although there is widespread agreement that multiple mechanisms that mediate pathogenesis likely operate simultaneously in the affected patient [68]. A potentially important clue toward the mechanism by which hantavirus infections deplete blood platelets and, in some cases cause hemorrhagic manifestations, was advanced by the recent discovery that pathogenic hantaviruses are able to recruit platelets to adhere to endothelial cell surfaces, with β3 integrin used as a critical binding element [69].

(3) Pathogenic effects caused by the activities of specific viral macromolecules. We have reviewed some of the activities associated with the Gn, Gc and N, virally-encoded polypeptides in previous sections.

Testing models of pathogenesis can be done more effectively when there is an animal model that mimics key aspects of the disease. There is no such model that closely mimics HFRS, but animal models exist for both the asymptomatic carriage of PUUV and SNV by their native carrier rodents, the bank vole *Myodes glareolus* and the deer mouse *P. maniculatus*; as well as a Syrian hamster model using ANDV or the related Maporal virus from Venezuela, for which an HCPS-mimetic disease is observed [70–73].

The ANDV-Syrian hamster model has a number of features in common with the human disease, as well as some differences. Unlike the neurologic diseases that have been possible to elicit with HTNV, the hamster model for HCPS appears to be caused by capillary leak that results in pulmonary edema and the production of a pleural effusion with exudative characteristics. Typically the hamsters die between 11 and 14-d post-inoculation, reflecting a slightly accelerated incubation period in comparison to human infections. As with human HCPS, the microscopic examination of the lung reveals abundant fibrin deposition, thickened alveolar septa, and viral antigen expressed abundantly in the microvascular endothelium. ANDV-infected hamsters fitted with physiologic monitoring devices exhibited diminished pulse pressures, tachycardia, and hypotension that appear to closely mimic the shock that is believed to be the proximate cause of demise in patients who succumb to HCPS [65,74].

Compared to the human disease, ANDV-infected hamsters exhibit exceptionally high titers of live ANDV in their tissues, with much of the viral replication occurring in hepatocytes, which are spared in the human disease. Titers of live ANDV in some cases exceed 10^8^/g, whereas hantavirus isolates from human tissues have been notoriously difficult to obtain. Despite the universal occurrence of mildly-elevated hepatic enzymes in patients with HCPS, hepatic enzymes do not appear to be present at elevated levels in the blood of diseased hamsters even immediately before death [75].

### 3.2. Adaptive Immune Responses

The protracted incubation period associated with hantavirus disease gives the host considerable time to mount a mature immune response against the virus. Thus, in contradistinction to infections of comparable severity and related symptomatology associated with arenaviruses and filoviruses, hantavirus infections of humans are associated with antibody responses of significant titer by the time symptoms commence. Despite this observation, it appears to be possible that natural variation in individual neutralizing antibody responses among patients with SNV infections can be linked to disease severity, suggesting that administration of antiviral antibodies could prove effective therapeutically [76]. In the case of ANDV infection, new evidence has emerged indicating that the apparent clearance of the virus from the blood does not result in the complete removal of antigenic stimulus by the virus, suggesting that the virus may persist, perhaps in some as-yet undetermined immunologically privileged site [77].

A role for T cell-mediated pathological responses in HFRS and HCPS has been the source of speculation for a variety of reasons. The severity of SNV-associated HCPS may have made it more apparent that the onset of pulmonary edema, tachycardia and hypertension seemed to be all but universally temporally associated with the appearance of a spectrum of highly-activated cells of the lymphoid lineage in the peripheral blood. Cells with a close morphologic similarity to these “immunoblasts” were detected in the congested, heavy lungs of patients who came to autopsy, as well as in lymphoid organs and in the portal triads [63,78–80]. These observations led to speculation that some component of hantavirus pathogenesis could be linked to the appearance of antiviral T cells that could stimulate or contribute to the appearance of a “storm” of mediators and the associated capillary leak phenotype. Subsequent studies have borne out the expectation that a significant fraction of the immunoblast population in patients with HCPS are T cells with specificity for specific class I HLA-presented epitopes of viral antigens, including Gn, Gc and N [77,81–83]. Presumably, the antiviral activities of such cells, manifested in part through their elaboration of mediators in the affected interstitium, can contribute to the endothelial/capillary leak that lies at the heart of hantavirus pathogenesis.

Because early cases of HCPS often came to autopsy, it became possible to examine necropsied tissues for expression of cytokines. The study by Mori *et al.* (1999) revealed high relative expression of proinflammatory cytokines including TNFα, IL-1β, IL-6, providing evidence in favor of a “cytokine storm” model for pathogenesis [64]. The authors believed, based on the morphology of cytokine-secreting cells, that both monocytes and lymphocytes were contributing to the production of cytokines. That proinflammatory mediators are found in elevated levels in the plasma as well as the renal interstitium of patients with acute hantaviral illness has been recognized for some time as well [84,85].

While diagnosis of HCPS as well as HFRS is best accomplished with IgM serology, in the acute stage of SNV infection, RT-PCR can also be used if blood cells or blood clot are used instead of plasma or serum, where sensitivity even using nested PCR primers drops to about 70% [86–88]. In a facility at which many cases of HCPS are treated, the University of New Mexico medical center in Albuquerque, a diagnostic service has long been offered in which the patient’s hematologic findings are analyzed to establish the probability that a patient has HCPS. The combination of thrombocytopenia, elevated abundance of “immunoblast” lymphocytes, left-shifted polymorphonuclear cell population without strong morphologic evidence for their activation, and elevated hemoglobin or hematocrit values is highly specific for HCPS and allows clinicians the ability to put presumptive-HCPS patients on extracorporeal membrane oxygenation (ECMO), which is believed to have saved many patients from a lethal outcome [89].

## 4. Epidemiology and Epizootiology

### 4.1. Epidemiology and Transmission Mechanisms from Rodents to Humans

Human infection by hantaviruses is thought to follow contact with secretions or excretions produced by infected rodents. In the United States, 538 human infections by hantavirus were reported through late December 2009 [90], with New Mexico, Arizona and Colorado exhibiting the highest case-loads. While the prototypical central American hantavirus in central America was Rio Segundo virus of *Reithrodontomys mexicanus* from Costa Rica, the first human disease appeared some years later in Panama, where Choclo virus (CHOV) arose as the etiologic agent and is believed to be responsible for all known cases of HCPS. The fulvous pygmy rice rat *Oligoryzomys fulvescens* has been identified as the rodent reservoir [91]. In Panama, the first cases of HCPS, albeit with little or no evident cardiac involvement, were reported in 1999, and since then, 106 human infections have occurred with a 26% mortality rate [92]. Serosurveys of mammals in Mexico and Costa Rica have found anti-hantavirus antibodies [93–96], and seroprevalences ranging between 0.6 to 1.6% in human populations were reported despite the absence of known HCPS cases [97]. In South America, HCPS cases have been indentified in Argentina, Bolivia, Brazil, Chile, Paraguay and Uruguay, and evidence for human exposure to hantaviruses have also been reported in Venezuela [98] and Perú [99]. In southern South America, ANDV is the main etiologic agent with cases in Chile and Argentina reported since 1995. In Chile, 671 cases of HCPS due to ANDV have occurred during the period 2001–2009 [100]. Since 1995, more than 1,000 HCPS cases have been reported in Argentina [101]; in Brazil, approximately 1,100 HCPS cases have been identified between 1993 and 2008 [102]. Case-fatality ratios in those three countries have been similar, ranging from 30% (Argentina), 36% (Chile) and 39% (Brazil).

Hantavirus infections occur more frequently in men than women, although the male/female ratio is highly variable. For example, Panamanian communities showed a ratio of 55 men to 45 women [103], while in Chile the ratio is more biased to males (71%) [104]. In the Paraguayan Chaco the male-female ratio approaches 50% [105]. In North America, by December 2009 63% of case-patients were males [90]. All ethnic and racial groups seem to be susceptible to hantavirus infections, and the differences between certain groups (as indigenous and non-indigenous) are more likely correlated with the type habitat where the population resides (e.g., rural *versus* urban areas). In fact, rural communities account for the highest hantavirus incidences overall and are therefore at higher risk [92,105–111], although the importance of peridomestic settings as a major area of exposure has also been emphasized [112,113].

The main mechanism by which humans acquire hantavirus infection is by exposure to aerosols of contaminated rodent feces, urine, and saliva [114,115]. This can occur when humans reside in areas in close proximity to those that rodents inhabit, live in areas infested with rodents, or when rodents invade human settings, which are more frequent in rural habitats. There is a long history of human co-existence with rodents, raising questions about the apparent recent increases in hantavirus-related illnesses, especially HCPS. Other than an apparent association with El Niño southern oscillation (ENSO) events in some regions [116,117], the recent increases in incidence of HCPS do not seem to follow a readily-defined temporal or spatial pattern. However, some landscape features such as habitat fragmentation or human-disturbed areas may influence rodent population dynamics and impact viral incidence [118–121]. Despite the stochasticity associated with contraction of hantavirus infection, certain scenarios have been recognized as posing higher risk. Human activities in poorly ventilated buildings that aerosolize particulates that are then inhaled (*i.e.*, cleaning, shaking rugs, dusting) are frequently identified among patients admitted for HCPS [11,122]. Outdoor activities are thought to convey lower risk due to lability of hantaviruses to UV radiation and the presumed tendency to be dispersed in wind, although certain environmental conditions seem to maintain the virus for longer periods outside its natural host allowing for indirect transmission [123]. An alternative but uncommon route of virus transmission is by rodent bites [124–126]. Field workers handling mammals are potentially at higher risk of exposure with hantavirus infections, although when quantified through serosurveys the absolute risk appears rather slight [127]. A new study in Colorado suggests the possibility that a rodent bite may have been the proximate vehicle for outdoor transmission of SNV [128], which re-emphasizes the use of personal protective equipment during field work activities [129]. As a particular case within hantaviruses, person-to-person transmission has exclusively been documented for the South American Andes virus [130–135]. The identification of this transmission route has been made using both molecular tools and epidemiological surveys, but the mechanism of interpersonal transmission is not well established. Recent findings show that family clusters and specifically sexual partners share the greater risk of interpersonal transmission, although sexual transmission *per se* can be neither inferred nor refuted presently [130,135]. Interestingly, ANDV may also be shed by humans through other biological fluids such as urine [136], illustrating the particular properties that differentiate this virus from other hantaviruses. Although interpersonal transmission seems to be unique for ANDV, viral RNA of PUUV has been detected in saliva of patients with HFRS, and some patients with SNV-HCPS have viral RNA in tracheal secretions [88,137].

### 4.2. Rodent Reservoirs and Cross-species Transmission

Hantaviruses in the Americas are naturally hosted by rodents (Muridae and Cricetidae) as well as shrews (Soricidae) and moles (Talpidae) (Figure 1). Three shrew and one mole species have been reported to host hantaviruses and their pathogenicity for humans remains unknown [22,138,139]. At least 15 rodent species have been identified as carriers of different pathogenic hantaviruses, with some South American genotypes such as Castelo do Sonhos (CDSV) or Hu39694 only identified after human infections (Figure 1). Hantaviruses typically show high species-specificity and no intermediate host [140]. However, some hantavirus genotypes have been described in the same rodent species. Such is the case of Playa de Oro (OROV) and Catacamas (CATV) identified in *Oryzomys couesi* [141,142], or Maporal (MAPV) and Choclo (CHOV) hosted by *O. fulvescens* [91,143]. In North America both Muleshoe and Black Creek Canal hantaviruses have been detected in geographically-distant *Sigmodon hispidus* [144,145]. Also, one hantavirus genotype (e.g., Juquitiba-like virus) may be carried by more than one rodent species (*O. nigripes, Oxymycterus judex, Akodon montesis*). Another example is Laguna Negra virus (LANV) which after being identified in *Calomys laucha* [146] has also been reported in *C. callosus* [147]. The rapid increase in the discovery of new hantaviruses and the identification of their hosts does not seem likely to end soon as new small mammal species are screened [95]. This subject is complicated by continued controversy in the criteria for the classification of distinct hantaviruses [148,149], which is also tied to host taxonomic classification and taxonomic rearrangements.

Cross-species transmission is a major process during spread, emergence, and evolution of RNA viruses [6,150]. Particularly within hantaviruses, spillover to secondary hosts are increasingly identified as more extensive studies are performed [151–156]. For example, ANDV is the predominant etiologic agent of HCPS in South America, and *O. longicaudatus* the main rodent reservoir. Spillover in at least four other rodent species that co-occur with the reservoir have been identified, with *Abrothrix longipilis* showing the second higher prevalence to ANDV-antibodies, and there is presently no question that the virus is extremely similar genetically between the two host rodents [157,158]. In North America, spillover of Bayou virus (BAYV) may have occurred from the main reservoir *O. palustris* to *S. hispidus*, *R. fulvescens*, *P. leucopus*, and *B. taylori* [159–161]. Hantavirus spillover is more likely to occur with host populations inhabiting sympatric or syntopic regions [151,162], and cross-species transmission would presumably have greater chances of success if the host species are closely related [163]. An interesting exception is found between Oxbow virus (OXBV) and Asama virus (ASAV) in which a host-switch process seemed to have occurred between mammals belonging to two families (Talpidae and Soricidae), likely as a result of alternating and recurrent co-divergence of certain taxa through evolutionary time [138].

### 4.3. Outbreaks

Hantaviruses are horizontally transmitted between rodents and are not transmitted by arthropods (unlike other viruses of the family Bunyaviridae). Spillover infection to nonhuman mammals usually results in no onward (or “dead-end”) transmission, but if humans are infected may result in high morbidity and mortality [122,164]. During the spring of 1993, an outbreak of patients with HCPS due to SNV occurred in the Four Corners states resulting in more than 60% case-fatality among the initial cases, many involving members of the Navajo tribe [12,121]. In Panama, an outbreak was reported during 1999–2000 in Los Santos, and 12 cases where identified with three fatalities [165,166]. This represented the first report of human hantavirus infections in Central America. In South America, the first largest identified outbreak occurred in the Chaco region in northwestern Paraguay during 1995–1996. Seventeen individuals were identified with SNV antibody (ELISA) or were antigen (IHC) positive out of 52 suspected cases [167]. Major outbreaks due to ANDV occurred in 1996 in southern Argentina [131,134]; in southern Chile clusters of patients presented with hantavirus illness in 1997 [158]. In Brazil, the first outbreak was identified in the Brazilian Amazon (Maranhão State) in 2000, and involved small villages that resulted in a 13.3% prevalence of those tested (398 total residents) [168].

The factors that trigger hantavirus outbreaks are still poorly understood, probably because they result from several interacting biotic and abiotic features whose key parameters are difficult to model. However, the use of new modeling approaches that involve geographical and environmental features seem to be promising in predicting potential hantavirus outbreaks and/or areas of higher risk [169–172]. Because hantaviruses are known to be directly transmitted from infected to susceptible hosts, the first natural approach is to relate outbreaks to the ecology of the viral hosts. Hantavirus transmission and persistence in rodent populations depends on several factors that interact to affect ecological dynamics of the host, which in turn is strongly influenced by the behavioral characteristics of individual rodent species, to landscape structure, and environmental features [173,174]. Viral transmission depends on contact rates among susceptible hosts, and despite the prevailing notion that a higher density increases encounters and hence secondary infected hosts, contrasting patterns relating rodent population size and virus prevalence can be found [175]. In addition, it has been shown that SNV transmission follows a contact heterogeneity pattern, where individuals in the population have different probability of transmitting the infection [176]. The understanding of viral transmission proves to be far more complex when species other than the main reservoir host are incorporated in the model. In fact, recent studies have shown that higher hosts species diversity is correlated with lower infection prevalence in North America for *P. maniculatus* [177], in Central America for *O. fulvescens* (reservoir of Choclo virus) and *Zygodontomys brevicauda* (reservoir of Calabazo virus) [178], and in South America for *Akodon montensis* (reservoir of Jabora virus) [162]. Contact rates vary according to the spatial distribution of populations and seem to be strongly influenced by landscape structure. For example, SNV prevalence in *P. maniculatus* was higher in landscapes with a higher level of fragmentation of the preferred habitat [179]. In addition, certain properties of the landscape such as elevation, slope, and land cover seem to be useful in detecting areas with persistent SNV infections, and therefore thought to be refugial areas where the virus can be maintained for years [169]. Changes in the natural environment of reservoir species, such as forest fragmentation and habitat loss, may alter population abundance and distribution and lead to hantavirus outbreaks, as observed in the Azurero Peninsula of Panama [118,119]. Also, differences in the microhabitat, including overstory cover, may lead to differences in the ecological dynamics within populations and affect the rate of exposure to the virus [180]. Differences in hantavirus infections through contrasting landscapes in the latitudinal span have been found in rodent populations of *O. longicaudatus* in Chile, suggesting that humans are differentially exposed to the virus [107,181].

Rodent population dynamics are affected by seasonal changes of weather and climate [182,183]. In the case of the ENSO-associated outbreaks, a complex cascade of events triggered by highly unusual rains in the precedent year have been postulated to result in an increase of primary production and rodent densities, also increasing the likelihood of transmission of the virus to humans, but it has proved difficult to precisely demonstrate the suggested intermediate events such as increased rodent densities in the increased caseload [116,121,184]. In South America, effects of climate change and hantavirus outbreaks have not been well studied, despite the knowledge that several rodents species that are reservoirs of emerging diseases have dramatically been affected by events like El Niño [185]. Changes in host population dynamics are also affected by seasonality, which may lead to disease outbreaks when processes that equilibrate rodent populations from season to season are interrupted [186].

Viral emergence may continue to be promoted as human-introduced changes continue to increase in the environment at different geographical scales. Human incursions into previously uncultivated environments may lead to new contacts between rodent reservoirs and humans, increasing the likelihood of contracting infections [187]. These changes may also alter rodent’s population structure and dynamics and interspecies interactions creating conditions that may lead to viral outbreaks, viral establishment in new hosts, and emergence of HCPS [102,162], even with seemingly slight ecological disturbance to the virus-host system [188].

## 5. Concluding Remarks

Certain pathophysiologic characteristics, including thrombocytopenia and shock, of hantavirus diseases of humans, bear substantial similarity to the hemorrhagic fevers induced by other viruses such arenaviruses, filoviruses and flaviviruses, despite sharing essentially no sequence similarities therewith. Such observations raise questions about whether such commonalities in pathogenesis are chance similarities of phenotype, or instead report the presence of common molecular mechanisms among the viruses.

In this review we discuss the general properties, discoveries and epidemiology/ecology of the New World forms of pathogenic hantaviruses, and also seek to identify some of the characteristics of the viral macromolecules and immunologic mechanisms that have been proposed as potential direct mediators of the pathogenic events that characterize the human disease HCPS. While it is unlikely that expression of any particular viral protein or RNAs in isolation can be relied upon to replicate key phenotypes of infection by the complete virus, some of the findings have been sufficiently consistent with what is known of the pathogenesis *in vivo* that they offer plausible first-pass leads in the search for therapeutic targets. We look forward to the mechanistic revelations that will follow the inevitably expanded usage of powerful methods such as deep sequencing, ever-more advanced imaging, and microscopic methods, and animal models that can at last be said to be close mimics of human hantavirus disease.

## Figures and Tables

**Table 1:** Major human-pathogenic hantaviruses.

	Virus strain	Acronym	Geographic Distribution	Host	Pathology	Case-fatality ratio (%)
New World	Sin Nombre	SNV	North America	*Peromyscus maniculatus*	HCPS	−35
	Choclo	CHOV	Panamá	*Oligoryzomys fulvescens*	HCPS	∼21
	Andes	ANDV	Argentina, Chile	*Oligoryzomys longicaudatus*	HCPS	35
	Laguna Negra	LANV	Argentina, Bolivia, Paraguay,	*Calomys laucha, C. callosus*	HCPS	5–15
Old World	Hantaan	HTNV	China, Korea, Russia	*Apodemus agrarius*	HFRS	5–0
	Seoul	SEOV	Worldwide	*Rattus rattus, R. norvegicus*	HFRS	1–2
	Puumala	PUUV	Scandinavia, western Europe, Russia	*Myodes glareolus*	HFRS/NE	0.1–0.4
	Dobrava-Belgrade	DOBV	Balkans	*Apodemus flavicollis*	HFRS	5–10

HCPS: Hantavirus cardiopulmonary syndrome; HFRS: Hemorrhagic fever with renal syndrome; NE: Nephropathia epidemica

**Table 2:** Similarities and differences between HFRS and HCPS.

	HFRS	HCPS
Transmission	Aerosolized wild-rodent excreta, no arthropod vector	Aerosolized wild-rodent excreta, no arthropod vector
Stages	Incubation period: 1–5 wk, prodrome 5–10 d, hypotensive phase 1–3 d oliguric phage 3–5 d convalescence	Incubation period: 1–5 wk, prodrome (febrile phase) 3–10 d, cardiorespiratoryphase 1–6 d diuretic phase 1–3 d convalescence
Primary organ of attack	Kidney, lymphoreticular system	Lungs, lymphoreticular system
Location of effusion	Retroperitoneal space	Thoracic bed; pulmonary interstitium
Pathologic hallmarks	Capillary leak. Mononuclear infiltrate.	Capillary leak. Interstitial mononuclear cell infiltrate.
Clinical findings	Fever, chills, nausea and vomiting, headache, lethargy, oliguria, tachycardia, facial and truncal flushing, hemorrhage, occasionally noncardiogenic pulmonary edema with tachypnea, shortness of breath, shock, death in 0.1 to 5%.	Fever, chills, nausea and vomiting, headache, lethargy, dyspnea, tachycardia, tachypnea, shortness of breath, noncardiogenic pulmonary edema, hemorrhage/petechiae (ANDV), shock, death in 10 to 50%.
Laboratory findings	Hypoalbuminemia, azotemia, proteinuria, hematuria, leukopenia/leukocytosis	Hypoxia, Hypoalbuminemia, thrombocytopenia, leukopenia/leukocytosis, hemoconcentration
Treatment	Specific: ribavirin. Nonspecific: cardioventilatory support, dialysis	Specific: none. Nonspecific: extracorporeal membrane oxygenation (ECMO), cardioventilatory support
